# Mechanosensitive potassium channels in neurons projecting cardiac axons of the nodose ganglion in rats

**DOI:** 10.3389/fphys.2025.1644488

**Published:** 2025-10-08

**Authors:** Peter Linz, Eva Hutter, Tillmann Ditting, Mario Schiffer, Kerstin Amann, Karl F. Hilgers, Roland Veelken, Kristina Rodionova

**Affiliations:** ^1^ Department of Internal Medicine 4, University of Erlangen-Nürnberg, Erlangen, Germany; ^2^ Department of Pathology (Section Nephropathology), Friedrich-Alexander University Erlangen, Erlangen, Germany

**Keywords:** mechanosensitive potassium channels, cardiac baroreflex, vagal afferent, nodose ganglion, neuron

## Abstract

Cardiac vagal afferent neurons, located in the nodose ganglion, play a pivotal role in cardiopulmonary reflexes that link cardiac filling states to renal sympathetic outflow and the maintenance of circulatory homeostasis. Their excitability depends on a fine balance of depolarizing and repolarizing ion fluxes, yet the contribution of mechanosensitive (MS) ion channels to this regulation remains incompletely understood. While non-selective cation channels such as Piezo1/2 are established mediators of baroreceptor function, they are not directly responsible for repolarization. In contrast, mechanosensitive potassium channels are ideally suited to terminate action potentials and thereby shape afferent signaling from the heart. We, therefore, tested the hypothesis that MS potassium channels are functionally expressed in nodose ganglion neurons with cardiac projections. Using excised-patch recordings with stepwise suction, we identified two types of MS channels. One was inhibited by extracellular gadolinium (100 µM) and exhibited a higher unitary conductance, while the other was insensitive to gadolinium and showed a lower conductance. Both channel types were predominantly selective for K^+^ but also permeable to Na^+^, with a relative K^+^: Na^+^ permeability of ∼3.3–3.4. This mixed selectivity provides sufficient depolarization to activate voltage-gated Na^+^ channels and thereby initiate action potential firing. Our findings provide direct evidence for the presence of MS potassium channels in cardiac vagal afferent neurons and suggest that they may contribute critically to the mechanoelectric coupling and reflex control of cardiovascular function.

## Introduction

The regulation of renal function is strongly coupled with cardiac sensory input (Feng et al., 2025; Johns, 2013). Stretch-sensitive receptors located in the atrial walls and great vessels detect changes in cardiac filling and wall tension and transmit this information via afferent neural pathways to central autonomic control centers (van Weperen and Vaseghi, 2023). Through this cardiopulmonary afferent signaling, the heart exerts a profound influence on renal sympathetic nerve activity and thereby modulates sodium and water excretion (Evans and Bie, 2016). In the setting of structural cardiac remodeling—such as in heart failure without overt systolic dysfunction (Hamo et al., 2024)—this reflex arc may become impaired, leading to dysregulated renal sympathetic outflow and inappropriate fluid retention (DiBona et al., 1996).

We recently demonstrated that in *heart failure without overt systolic dysfunction*, the regulation of sympathetic renal nerve activity via cardiac afferent pathways is impaired (Pickny et al., 2023). This disturbance appears to originate from dysfunctional sensory nerve endings embedded in the altered myocardial tissue (Chapleau et al., 2001; Moore, 2024; Pauziene et al., 2023). Primary afferent neurons located in the nodose ganglion, which relay signals from the heart, displayed increased excitability and elevated firing rates *in vitro*. However, this enhanced neuronal responsiveness did not restore afferent-mediated reflex function *in vivo*, suggesting that other mechanisms—beyond simple excitability—are involved in the breakdown of afferent control.

Cardiac afferents are predominantly unmyelinated C-fibers with chemo- and mechanosensitive (MS) properties (Veelken et al., 2003), and their excitability depends on the coordinated activity of ion channels mediating depolarization and repolarization. Although voltage-gated sodium channels such as Nav1.7 and Nav1.8 initiate action potentials (Catterall, 2023; Vasylyev et al., 2024; Ditting et al., 2016; Kwong et al., 2008), they are not the primary focus of the present study. Rather, we concentrated on the repolarization phase, which is shaped largely by potassium conductance and may be influenced by mechanical stimuli. MS potassium channels are strong candidates for dynamically adjusting afferent excitability in response to changes in cardiac wall tension.

Despite MS Piezo1 and Piezo2 channels having emerged as highly prominent targets of research (Lacroix and Wijerathne, 2025; Wang and Xiao, 2018) and having advanced our understanding of cardiovascular mechanotransduction and baroreceptor signaling (Zeng et al., 2018; McGrane et al., 2025), these non-selective cation channels primarily conducting calcium play only an indirect role in neuronal repolarization. In contrast, potassium channels activated by mechanical stress are directly suited to terminate action potentials and reset neuronal excitability.

However, while Piezo channels are an essential element for mechanoelectrical coupling, their role in neuronal repolarization is likely indirect. The role of Ca^2+^-sensitive potassium channels in action potential generation is considered modulatory in terms of neuronal excitability (Orfali and Albanyan, 2023). In contrast, potassium channels activated by mechanical stress are directly suited to terminate action potentials and reset neuronal excitability (Kanda et al., 2023). This distinction underlies the rationale of the present study, which focuses on identifying and characterizing MS potassium channels as potential key modulators of cardiac afferent function.

The present study was, therefore, designed to test whether MS potassium channels are functionally expressed in nodose ganglion neurons with cardiac projections. We used whole-cell patch-clamp recordings from primary cultures of nodose ganglion neurons and applied gadolinium—a known blocker of stretch-activated ion fluxes (Zhang et al., 2018)—to assess the presence and functional contribution of stretch-sensitive potassium conductance in these neurons. We focused specifically on cardiac afferent neurons because of their central role in modulating renal sympathetic nerve activity via cardiopulmonary reflex pathways (Feng et al., 2025; Johns, 2013; van Weperen and Vaseghi, 2023). Hence, a special method was used, as previously described, to identify neurons with cardiac axons in the nodose ganglion (Pickny et al., 2023). Understanding how cardiac sensory neurons process mechanical input is essential for elucidating the mechanisms that couple cardiac filling states to renal sodium and water excretion.

## Methods

For the experiments, male Sprague–Dawley rats (Ivanovas, Kisslegg, Germany) weighting 250 g–300 g were maintained in cages at 24 ± 2 °C. They were fed a standard rat diet (no. C 1000, Altromin, Lage, FRG) containing 0.2% sodium by weight and were allowed free access to tap water. All procedures performed on the animals were carried out in accordance with the guidelines of the American Physiological Society and in compliance with the NIH Guide for Animal Care and Use in Laboratory Practice. They were approved by the local government agency (Regierung von Unterfranken). We retrogradely labeled cardiac neurons in twelve rats. The preparation failed in 5% of the animals. A total of 753 successful excised inside-out patch recordings out of a total of 820 patches were included in the data analysis.

### Retrograde labeling of cardiac neurons

To identify cardiac baroreceptor neurons, these cells were labeled via intrapericardial application of the dicarbocyanine dye 1,1′ dioleyl-3,3,3′ tetramethylindocarbocyaline methansulfonate (DiI) (D9–DiI, Molecular Probes, Eugen, OR) at the junction of the great vessels with the heart in male Sprague–Dawley rats (4–8 weeks old, Ivanovas, Kisslegg, Germany), as previously described (Pickny et al., 2023; Linz and Veelken, 2002). Animals were anesthetized. Mechanical ventilation was established via a tracheal tube, and a high midline thoracotomy was performed. The lobes of the thymus were carefully separated from each other, exposing a small portion of the roof of the pericardial sac adherent to the thymus. The roof of the pericardial sac was slightly opened to insert a fine glass cannula filled with DiI for respective intrapericardial application (10 µL of DiI 50 mg/mL). Afterward, the roof of the pericardial sac was closed by apposing the two lobes of the thymus and sealing them together with polyacrylic glue. Finally, the thorax was closed in layers (Pickny et al., 2023; Veelken et al., 1990). We allowed 1 week for DiI to be transported back to the neuronal cells in the nodose ganglion.

### Neuronal cell culture

Primary neuronal cultures of the nodose ganglia was established following previously described protocols (Pickny et al., 2023; Linz and Veelken, 2002; Ditting et al., 2003). Rats were anesthetized with hexobarbital. Both nodose ganglia were dissected and treated with a solution of collagenase (type 1A, 2 mg/mL, Sigma) in high-glucose Dulbecco’s modified Eagle’s medium (DMEM, Biochrom) on a stirring platform in a 5% CO_2_ incubator at 37 °C. After 1 h, ganglia were transferred into PBS solution containing trypsin (type III, 2 mg/mL, Sigma) and incubated for 10 min. Enzymatic activity was terminated using DMEM containing 10% FCS (Gibco, BRL). The softened tissue was triturated using siliconized pipettes. After centrifugation for 5 min, the supernatant was drawn off, and the cells were resuspended in a medium containing high-glucose DMEM, 10% FCS, and penicillin/streptomycin. The cells were cultured on poly-L-lysine-coated coverslips for 6–7 days until they were used for the studies.

We used only DiI-labeled neurons of the nodose ganglion that were unequivocally of cardiac origin. For this purpose, a small laser beam (a wavelength of 532 nm) powered by a storage battery was mounted to the patch-clamp recording setup. This equipment allowed for the detection of DiI-stained nodose ganglion cells, facilitating the identification of proper neurons for single-channel recordings.

### Single-channel recordings

Cells were transferred into a 1-mL recording chamber. The bath solution contained (in mM) 140 KCl, 1 MgCl_2_, and 10 HEPES. The pH was adjusted to 7.35 with KOH. In some experiments, the concentration of chloride was reduced by the use of potassium sulfate instead of potassium chloride to identify chloride conductances. Sodium conductances were identified by substituting KCl with NaCl in both the extracellular and intracellular solutions. Patch pipettes had resistances of 4 MΩ–5 MΩ and were filled with a solution containing (in mM) 140 KCl, 2 CaCl_2_, 1 MgCl_2_, and 10 HEPES. The pH was adjusted to 7.35 with KOH. Tetraethylammonium chloride (TEA, 40 mM) was used to block voltage-activated currents, especially potassium-activated currents. The trivalent lanthanide salt gadolinium chloride (100 µM) was used to inhibit MS-channels (see Zhang et al., 2018; Hamill and McBride, 1996).

The activity of MS channels was recorded from excised inside-out patches using an Axopatch 200B Patch-Clamp Amplifier (Axon Instruments, California) and low-pass filtered at 2 kHz. Seal resistances ranged from 10 GΩ to 20 GΩ. The membrane was voltage clamped at −60 mV, which is the typical resting membrane potential for the types of neurons investigated (Bobryshev et al., 2012; Ikeda et al., 1986). To obtain the current–voltage (I–V) relationship from each channel, the voltage was stepwise increased from −100 to +40 mV, and channel currents were measured.

In eukaryotic systems, Piezo and TRP channels exhibit distinct biophysical properties that allow for clear differentiation—such as differences in unitary conductance and ion selectivity (Coste et al., 2012; Nilius and Honoré, 2012). Hence, the classification of channels found in our experiments was based on those well-established, previously described single-channel parameters.

### Application of suction

To activate MS channels, suction was applied to the pipette holder. A mercury-calibrated manometer was used to monitor pressure steps, which were achieved using a syringe. The steps reached their plateau and remained stable within 0.2 ms. They ranged from 0 to 60 mmHg negative pressure. The change per step was 20 mmHg, and each step was held for 10 s. After activation, the channels were recorded once more at 0 mmHg to demonstrate the release properties of the openings.

### Change of solutions

In some experiments, the pipette solution was exchanged using the pipette-perfusion technique (Tang et al., 1990). For this purpose, a thin glass perfusion pipette (diameter 100 µm) with a tip opening of approximately 10 µm was placed within the patch-pipette at a distance of 50 µm–100 µm away from the patch. The distance was necessary to prevent disruption caused by the perfusion stream. At a perfusion pressure of 100 mmHg, the new solution reached the patch within several hundred microseconds, as demonstrated by the blockade of TEA-sensitive potassium channels perfused with 40 mM TEA. Pharmacological agents can be washed out with this method within several seconds. To prevent an out-streaming of the solution during the suction protocol, the perfusion pipette was only placed within the patch pipette when it was used immediately before and after the inside-out measurements. When MS- and voltage-gated potassium-channels were found simultaneously in a patch, TEA-block was used to study MS-conductances.

### Data analysis

Recordings were digitized at 5 kHz and analyzed off-line using pCLAMP software (version 8.0.1., Axon Instruments).

In all cases within a single patch, we reported the product NP_o_, where N is the number of channels and P_o_ is the open probability of a single channel. This approach is the standard in single-channel analysis when the exact number of channels in a patch cannot be determined with certainty (England et al., 1993). Across a large number of studies (Kosakai et al., 2015; Dove et al., 1998; Shoemaker and Worrell, 1991; Gu et al., 2014; Kolesnikov et al., 2021; Meng et al., 2022), NP_o_ provided a consistent, quantitative index of how experimental manipulations (alternative nucleotides, kinases, second messengers, mechanical forces, pharmacological agents, disease states, or genetic variants) alter the activity of single ion channels. Because NP_o_ inherently incorporates both the number of channels in a patch and their individual gating behavior, it is especially well-suited to reveal changes in open probability, frequency of openings, lifetime of open or closed states, and the recruitment of previously silent channels.

Since N was not directly accessible in excised-patch recordings without further manipulations (e.g., non-stationary noise analysis or full activation protocols) in our experiments, and such procedures were not feasible in our experimental paradigm, we chose the NP_o_ metric as described above as the most reliable and interpretable parameter (for more details, see below). This allows for meaningful comparisons across conditions without requiring assumptions regarding the absolute number of channels.

To express the activity of MS channels, the product *NP*_*o*_ was calculated, where *N* represents the number of single MS ion channels in the patch and *P*_*o*_ represents the open probability of each channel. *P*_*o*_ cannot be expressed directly, as outlined above, because the number of active channels in patch-clamp-experiments is not known. *NP*_*o*_ was determined by the events list method. Each opening and closing event was identified, and the sum of the open intervals was divided by the total duration of the recording period.

Negative currents represented a flow of positive charges from the inside to the outside of the membrane according to the general convention. All data are expressed as the means ± SEM, unless otherwise stated. Data were compared using ANOVA and respective post hoc tests. A value of *p* < 0.05 was considered statistically significant.

## Results

### MS channel activity in cardiac nodose neurons

**FIGURE 1:**
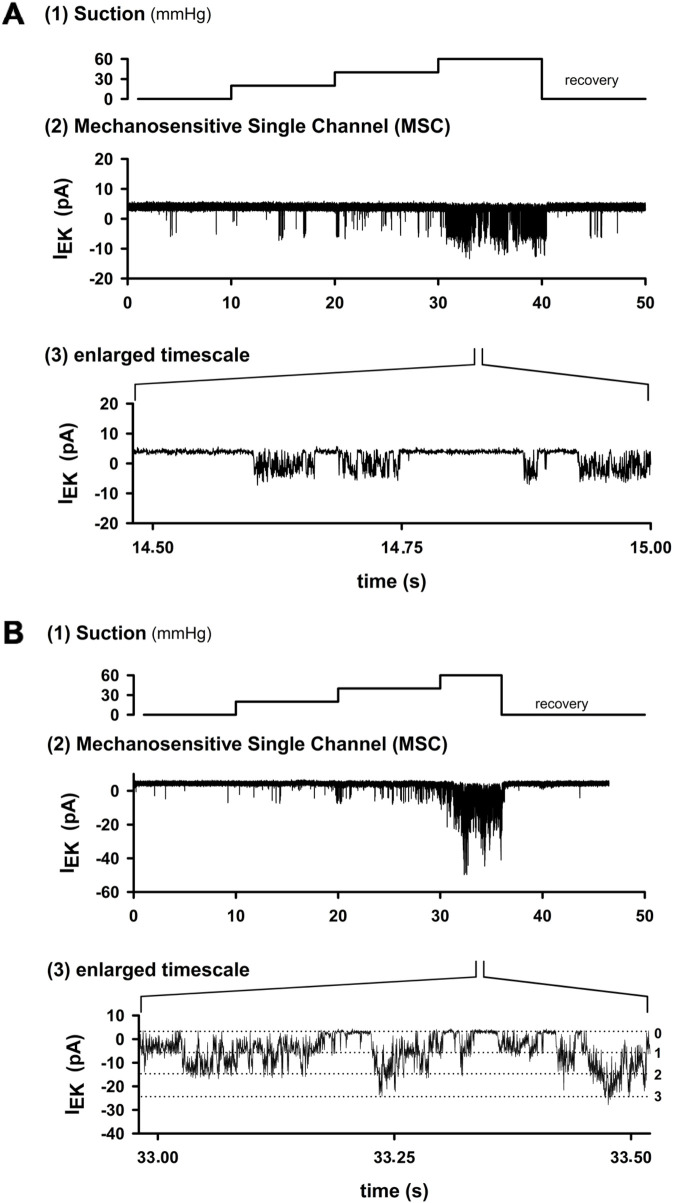
Activation of MS channels in cardiac nodose neurons. **(A)** Example trace from an excised inside-out patch. Stepwise increases in negative pipette pressure (10 s per step) evoked channel openings, which returned to baseline after suction release. At higher resolution, channel activity showed rapid “flickering” openings typical of MS channels. **(B)** Another patch with channels of smaller amplitude but higher overall activity (higher NP_o_). Three distinct conductance levels can be resolved on an expanded timescale. Hence, mechanosensitive channels were observed in 28.8% of patches under 2 mM Ca^2+^ in the pipette (134/332) and only 3.7% of patches under EGTA (16/421). These data demonstrate that cardiac nodose neurons express stretch-activated ion channels whose activity increases with mechanical stimulation.

To determine whether cardiac-projecting vagal sensory neurons express MS ion channels, we performed a total of 753 excised inside-out patch recordings: 332 under control conditions with 2 mM Ca^2+^ in the pipette and 421 under Ca^2+^-free conditions (10 mM EGTA). The application of negative pipette pressure elicited distinct single-channel openings that disappeared immediately after the release of suction (Figure 1A). Channel activity increased stepwise with rising suction and, on the expanded timescale, displayed the rapid “flickering” openings that are characteristic of MS channels (Figure 1B). In some patches, channels of smaller amplitude but higher overall activity (NP_o_) were detected, often with multiple distinct conductance levels resolved (Figure 1B).

MS channel activity was detected in 134/332 patches (28.8%) under control conditions, while the remaining 198 patches (71.2%) showed no mechanosensitivity. In contrast, under Ca^2+^-free conditions (10 mM EGTA), activity was only detected in 16/421 patches (3.7%). Thus, the presence of MS channels in cardiac projecting nodose neurons depends strongly on intracellular Ca^2+^ buffering, and not all retrogradely labeled neurons contain these channels. These results provide the first direct evidence that this subset of vagal afferents possesses membrane channels that can be directly activated by mechanical stretch.

**FIGURE 2:**
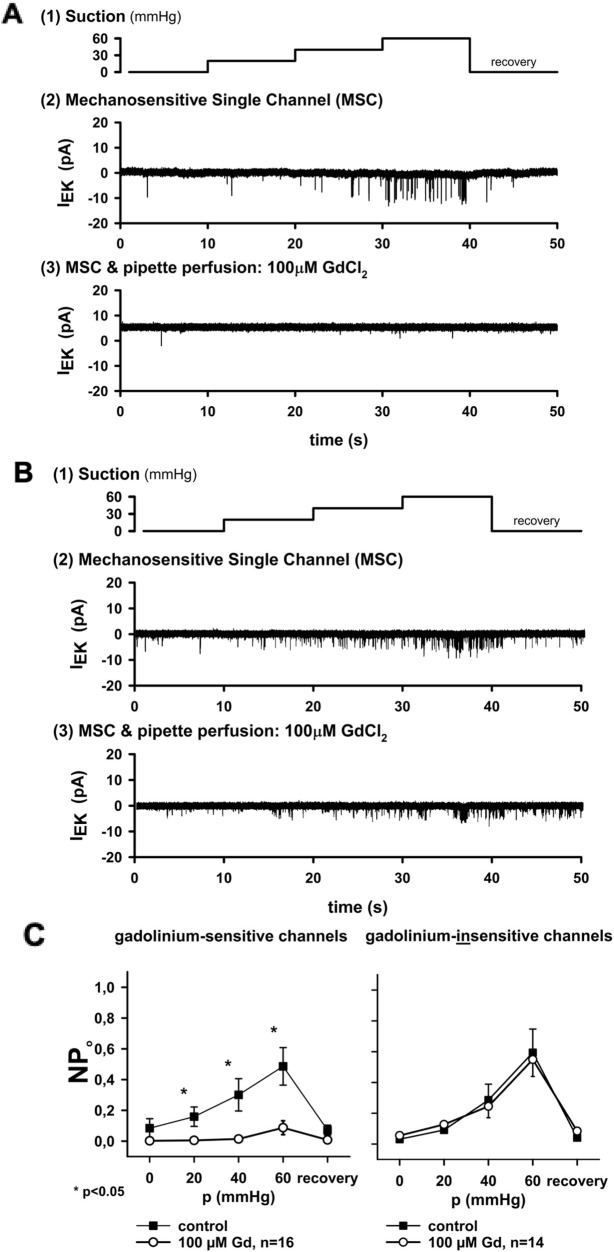
Gadolinium distinguishes two types of MS channels. **(A)** In one group of channels, activity was strongly inhibited by extracellular 100 μM Gd^3+^, a known blocker of MS currents. **(B)** In another group, channel activity was unaffected by Gd^3+^. **(C)** Summary plots of open probability (NP_o_) versus applied pressure. Inhibition by Gd^3+^ was significant in sensitive channels (n = 16, *p < 0.05) but not in insensitive channels (n = 14). Thus, two distinct channel populations could be differentiated in cardiac nodose neurons: Gd^3^+-sensitive and Gd^3^+-insensitive. Conductance analysis was only performed in the open (i.e., unblocked) state of the channel.

**FIGURE 3:**
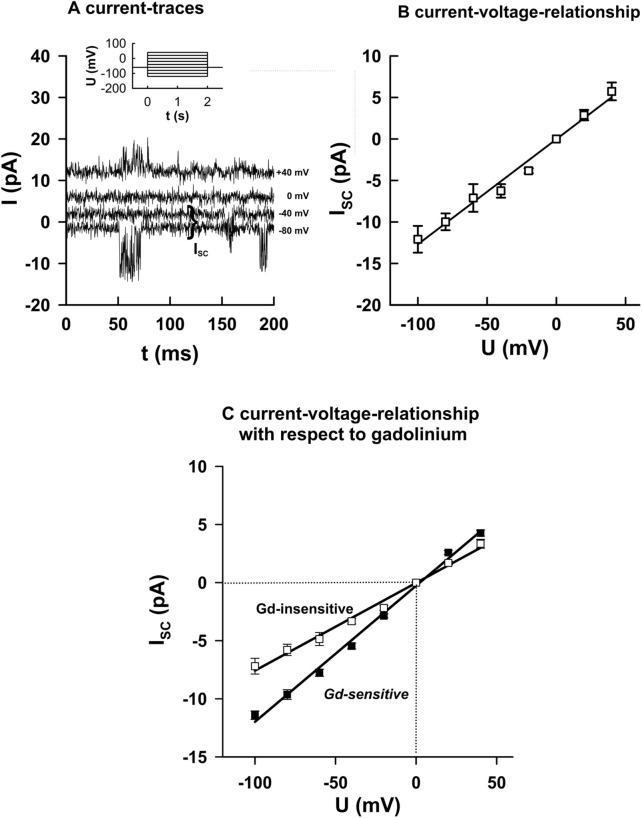
Biophysical properties of Gd^3+^-sensitive and -insensitive MS channels. **(A)** Example of currents recorded during voltage steps (−100 to +40 mV, 2 s duration) under constant suction (20 mmHg–40 mmHg). **(B)** Corresponding current–voltage relationships from individual openings (mean ± SEM, 10 events each). **(C)** Summary data: Gd^3+^-sensitive channels exhibited higher slope conductance (116.4 ± 5.3 pS, n = 16) than Gd^3+^-insensitive channels (79.1 ± 6.5 pS, n = 14; *p* < 0.05). Both groups reversed near 0 mV under symmetrical K^+^ conditions, indicating predominant K^+^ selectivity. These results identified two functional classes of MS channels differing in single-channel conductance. The slopes of the regression lines were significantly different from each other (*p* < 0.05).

**FIGURE 4:**
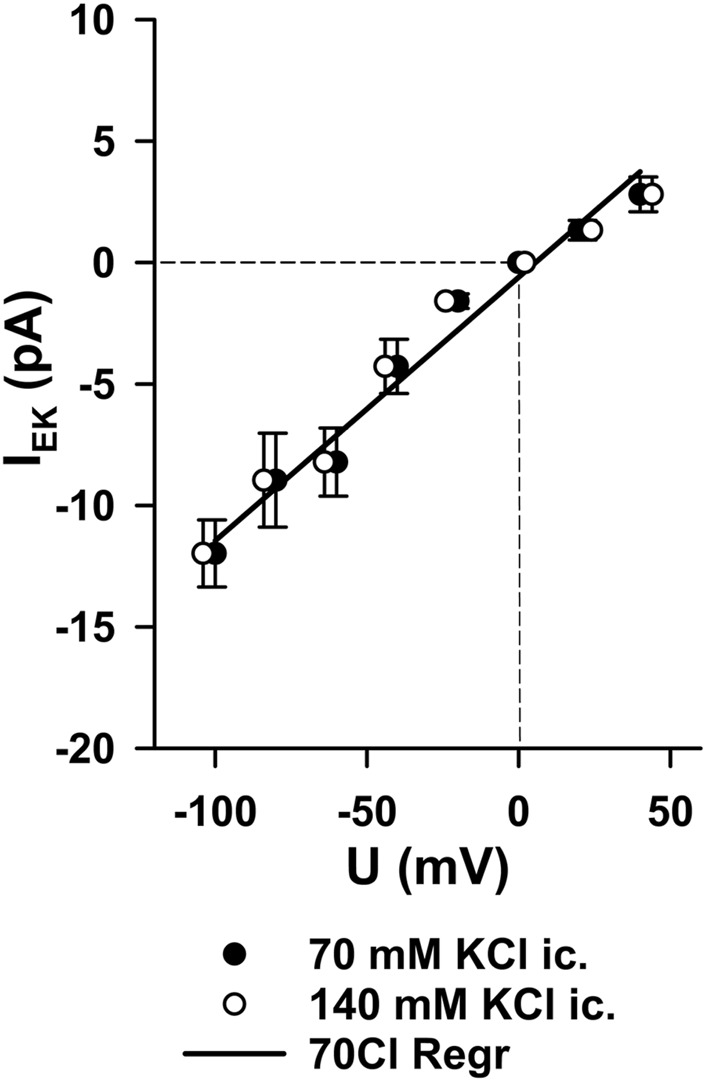
Lack of chloride permeability in MS channels. When extracellular chloride was replaced by sulfate, the current–voltage relationship of MS channels was unchanged. Reversal potentials remained close to 0 mV in both Gd^3+^-sensitive channels (n = 3) and Gd^3+^-insensitive channels (n = 3). This confirms that the identified MS channels conduct mainly cations and exclude Cl^−^ as a significant permeant ion.

In patch-clamp experiments, inward cation currents are defined as negative currents. Opening properties of the MS channels—including voltage dependence and conductance—are comprehensively analyzed and presented in Figures 2–4, where representative single-channel traces and quantitative summaries are displayed.

### Two MS channel populations defined by gadolinium sensitivity

We next tested whether MS channels in cardiac nodose neurons could be distinguished pharmacologically by the sensitivity to gadolinium (Gd^3+^), a broad inhibitor of MS currents. Of the 30 patches tested with 100 μM Gd^3+^, 16 were strongly inhibited, while 14 were unaffected (Figures 2A, B). Group data confirmed a significant reduction in open probability (NP_o_) in the sensitive group (*p* < 0.05), whereas no effect was observed in the insensitive group (Figure 2C).

Importantly, the maximum NP_o_ values differed between the two groups: 0.26 ± 0.05 in Gd^3+^-sensitive channels versus 0.48 ± 0.07 in Gd^3+^-insensitive channels (*p* < 0.05). This indicates that gadolinium not only blocks one population but also distinguishes channels with different activity profiles.

### Biophysical properties distinguish Gd^3+^-sensitive and -insensitive MS channels

To further characterize the two channel populations, we examined single-channel currents during voltage steps from −100 to +40 mV under constant suction (Figure 3A). At negative potentials, channel openings carried inward currents, while at depolarized potentials, outward currents were observed.

I–V relationships were constructed from individual openings (Figure 3B). Gd^3+^-sensitive channels exhibited a significantly higher slope conductance (116.4 ± 5.3 pS, n = 16) than Gd^3+^-insensitive channels (76.1 ± 6.5 pS, n = 14; *p* < 0.05; Figure 3C). Both groups reversed close to 0 mV under symmetrical K^+^ conditions, confirming predominant K^+^ selectivity. These data show that the two MS channel populations differ not only in pharmacology and open probability but also in their single-channel conductance.

### MS channels do not conduct chloride ions

To test for Cl^−^ permeability, extracellular chloride was replaced with impermeant sulfate. The I–V relationship and reversal potential of MS channels were unchanged in both Gd^3+^-sensitive (n = 3) and Gd^3+^-insensitive channels (n = 3; Figure 4). This excludes chloride as a relevant permeant ion and confirms that both channel populations predominantly conduct cations.

According to standard electrophysiological convention, inward cation currents (flow of positive charge into the cell) are plotted as negative currents. In our recordings, this refers to single-channel openings at negative membrane potentials. The linear I–V relationship refers to the voltage dependence of the current across a broader range, confirming the ohmic behavior of the channels without voltage gating. These observations are not contradictory but reflect the compatibility of ohmic linearity with inward currents at negative potentials, depending on the ionic driving forces.

### Sodium permeability of MS channels

**FIGURE 5:**
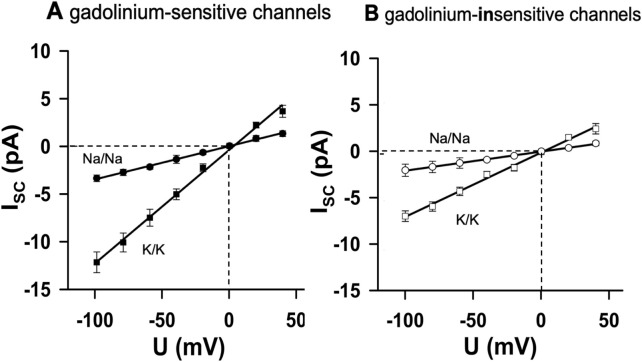
Sodium permeability of MS channels. Replacement of K^+^ with Na^+^ on both sides of the membrane reduced slope conductance in both channel types. **(A)** Gd^3+^-sensitive channels (n = 4). **(B)** Gd3+-insensitive channels (n = 4). The calculated permeability ratios of K^+^: Na^+^ were ∼3.3–3.4 in both groups (*p* < 0.05 between the regression slopes). Thus, cardiac nodose MS channels were predominantly K^+^ -selective but also allowed Na^+^ permeation, sufficient to depolarize neurons toward the threshold for action potential generation.

Finally, we examined whether the MS channels conduct sodium ions. When K^+^ was replaced by Na^+^ on both sides of the membrane, slope conductance was reduced in both channel types (Figures 5A, B). The calculated permeability ratios of K^+^: Na^+^ were 3.3–3.4 in both groups (n = 4 each, *p* < 0.05).

Thus, although both channel populations are predominantly K^+^-selective, they also permit Na^+^ entry. This mixed selectivity implies that the activation of MS channels by mechanical stimuli can depolarize cardiac-projecting nodose neurons toward the threshold for action potential initiation, thereby providing a plausible mechanism for mechanoelectric coupling in vagal afferents.

## Discussion

We hypothesized that neurons from the nodose ganglion with cardiac afferents should contain MS potassium channels that, among other effects, will support proper repolarization and post-hyperpolarization phases during action potential generation.

This paper could eventually demonstrate that in nodose ganglion neurons with cardiac afferents, there must exist at least two types of stretch-activated MS ion channels with predominant potassium conductance besides other proposed putative mechanotransducing mechanisms (Zeng et al., 2018). One exhibited a slope conductance of 116 pS and was blocked by 100 μM Gd^3+^, a known inhibitor of MS currents (Zhang et al., 2018). It has likely been described using cell-attached measurements previously (Hamill and Martinac, 2001). The author showed that in a co-culture with aortic endothelial cells, the number of patches on neurons containing these MS channels increased This points to a diffusible factor from endothelial cells, which might regulate the expression of these channels. The author was able to block MS activity when 20 µM of gadolinium was applied.

The other channel we could characterize was insensitive to extracellular application of 100 μM Gd^3+^ and had a smaller conductance of 76 pS. Interestingly, the channel with lower conductivity and, therefore, smaller channel width was insensitive to Gd^3+^, for the action of this substance is sometimes observed as mainly obstructing the pore. In a simple model, it would be easier to obstruct a small pore than a wide pore. Hence, not only the diameter but also the geometry or electrostatic properties of the pore may have an influence on the attachment and blocking effect of Gd^3+^. The insensitive channel shared properties of a 54 pS stretch-activated channel in colon sensory neurons (Su et al., 2000), which was also unaffected by 100 μM Gd^3+^, but in contrast to this channel, the MS channels of nodose neurons were not influenced by 40 mM TEA either on the extracellular side of the membrane.

Although gadolinium is considered a general inhibitor, several studies have shown that not all cellular mechano-transduction depended on Gd^3^-sensitive channels, so a classical Gd^3^ blockade of stretch-activated or MS cation channels failed to inhibit a mechanically evoked response (Sakamoto et al., 2010; Malek and Izumo, 1996; Steffensen et al., 1991).

Hence, gadolinium can help—as in our study—to distinguish between MS cation channels. The exact meaning of this finding is not yet understood.

Stretch-inactivated channels that were described in other preparations have not been observed at any patch on nodose neurons with cardiac afferents (Matsumoto et al., 2011). The existence of a mechanical notch filter that limits the range of the cell potential by two antagonizing channel types can, therefore, be assumed to be rather unlikely (Sackin, 1995a; Murthy, 2023).

The channels were predominantly selective for potassium and sodium and can, therefore, be classified as cation channels. In our measurements, the relative permeability of potassium versus sodium was 3.4 in Gd^3+^-sensitive channels and 3.3 in Gd^3+^-insensitive channels. Taking the Goldman equation into account with typical ion concentrations ([Na]_ec_/[Na]_ic_ = [K]_ic_/[K]_ec_ = 140 mM/5 mM), the reversal potential of the channels could depolarize the neurons to −33 mV, where voltage-sensitive sodium conductances are already activated.

Chloride or calcium conductances could not be detected in the two types of channels in our experiments. The calcium gradient used did not shift the reversal potential significantly from 0 mV. Therefore, the eventual permeability for this ion must be very low. Sharma et al. (1995) detected Gd^3+^-sensitive increases in intracellular Ca^2+^ of approximately 500 nM using the fura-2 technique when nodose neurons were mechanically stimulated. However, until now, it has not been possible to determine special membrane channels responsible for this increase.

When symmetrical potassium solutions were used, the two channel types showed the same reversal potential (0 mV) and a linear I–V relationship. The open probabilities (Bennett et al., 2000) were independent of voltage, suggesting that no voltage sensor is present in the channel proteins. So, they are unlikely to be influenced during the generation of action potentials in the neurons. Voltage insensitivity is a common feature of many MS ion channels in different preparations (Su et al., 2000; Sackin, 1995a).

The number of active MS channels in a patch decreased when extracellular calcium was buffered with EGTA. This suggests a new gating mechanism for neuronal MS channels as calcium normally acts from the intracellular side of the membrane on channel proteins, as observed in calcium-activated potassium channels. Extracellular Ca^2+^-dependent inhibition is known from the hair bundles in vertebrate hair cells, which are involved in the process of hearing. Here, mechanotransduction is abolished when reduced Ca^2+^ disrupts the tip links (Hamill and Martinac, 2001; Bennett et al., 2000). Calcium may modulate the structure of the channel protein, rendering it stretch-sensitive or inducing an active conformational state. On the other hand, EGTA (Kra et al., 1998) might be involved in the interaction itself.

The activity of MS ion channels in cardiac nodose neurons increased when the patch-clamp mode was changed from cell-attached to inside-out. Activation by the force of pipette retraction is not likely because microscopy analysis of patches showed purely fixed membranes (Hamill and Martinac, 2001; Gil et al., 1999). There is more evidence that membrane–cytoskeleton interactions are involved in the activation, as other authors have reported (Kra et al., 1998; Cunningham et al., 1997). Here, cytochalasin D and colchicine, which disrupted filaments of the cytoskeleton, are potent enough to increase channel activity in cell-attached patches (Lesage et al., 2000; Maingret et al., 1999). So, these channels may be inactivated by intracellular components in the parent cell.

The openings of nodose MS channels exhibited a flickering behavior, indicating an extremely low open-state time constant that could not be resolved by standard patch-clamp amplifiers. Based on amplifier specifications, the time constant must be faster than 0.1 ms. This limitation impacted both the accuracy of current measurements and the precise calculation of channel conductance. The flickering states were integrated into longer-lasting bursts on the millisecond timescale. In MS channels of chicken muscle cells, Guharay and Sachs (1984) showed that only this state was influenced by the cytoskeleton. Using cytochalasin, all other states remained unchanged (Sackin, 1995a; Sackin, 1995b).

Recent high-resolution electrophysiological studies have confirmed the presence of such rapid gating events in MS ion channels, emphasizing the need for advanced methods such as high-speed patch-clamp and fluorescence-based imaging to resolve these fast transitions (Cox et al., 2018; Wu et al., 2017; Jiang et al., 2021). The flickering states are typically integrated into longer bursts lasting several milliseconds.

In line with earlier findings, recent research confirms that cytoskeletal elements selectively influence particular gating states of MS channels. In particular, actin filament disruption using agents such as cytochalasin D alters prolonged open states, while rapid flicker transitions remain largely unaffected. These observations support a model in which mechanical force is transduced not only via membrane tension but also through tethering to the cytoskeletal network, particularly actin filaments (Jiang et al., 2021; Ingber, 2006).

In summary, the Gd-sensitive and -insensitive potassium channel types of cardiac nodose ganglion neurons shared many equivalent properties, with the exception of the conductivity. Further work is needed to elucidate the extent to which crosstalk between these channels and voltage-gated sodium channels influences the sensitivity and activity of cardiac afferent pathways in both healthy and diseased conditions.

Beyond the focus of this investigation, a large variety of stretch-activated channels (Webster et al., 2025) can be found in the nodose ganglion in general: Piezo1/2 were identified as directly stretch-activated cation channels essential for baroreceptor function, and their deletion abolished reflex-mediated activity (Zeng et al., 2018). In hypertensive rats, activation of Piezo1 by Yoda1 lowered the blood pressure through increased afferent firing, an effect prevented by GsMTx4, while SERCA2 inhibition attenuated these currents, indicating Ca^2+^-dependent gating (Cui et al., 2024; Zhao et al., 2025). TTN3 contributed to dynamic pressure sensing and baroreflex maintenance (Lu et al., 2020). Among TRP channels, TRPV4/TRPA1 were diet-sensitive, TRPC1/3 localized to low-threshold complexes, and TRPA1 and TRPV1 mediated visceral and gastro-esophageal mechanosensitivity, respectively. ENaC/ASIC2 deficits impaired baroreflex integration, while HCN channels, nitric oxide, and cytoskeleton-dependent stretch conductances further shaped mechanosensory excitability (Sharma et al., 1995; Kra et al., 1998; Li et al., 2016; Lu et al., 2009; Doan et al., 2004; Li et al., 1998). In addition to Piezo and TRP channels, members of the K2P family, such as TREK-1, have also been described in nodose ganglion neurons as MS potassium channels (Park et al., 2023). Although our data cannot exclude their contribution, the biophysical properties we observed (unitary conductance, Na^+^ permeability, and Gd^3+^ sensitivity) suggest that at least two distinct populations of MS K^+^ channels coexist. Future studies will be required to determine whether TREK1 or related K2P channels account for one of the populations described here.

A more definitive classification of the channels described in this publication would ideally involve genetic or structural analyses. Such experiments, however, were beyond the scope and feasibility of the present study. Instead, we adopted a functional classification approach based on comparisons with established mechanotransducers. Although this provides valuable insights into their properties, future studies employing genetic or structural methods will be required to confirm their molecular identity and further refine their mechanistic understanding.

## Data Availability

The original contributions presented in the study are included in the article/supplementary material; further inquiries can be directed to the corresponding author.
